# Supplementary material for: Dynamic sex chromosome expression in Drosophila male germ cells
Source: Nat Commun. 2021 Feb 9;12:892. doi: 10.1038/s41467-021-20897-y (PMC7873209; doi:10.1038/s41467-021-20897-y)
Supplement: Supplementary file 1 — Supplementary Information [file 41467_2021_20897_MOESM1_ESM.pdf]

Supplementary materials for Dynamic Sex Chromosome Expression in Drosophila Male Germ Cells

Sharvani Mahadevaraju*, Justin M. Fear*, Miriam Akeju*, Brian J. Galletta, Mara M.L.S. Pinheiro, Camila C. Avelino, Diogo C. Cabral-de-Mello, Katie Conlon, Stafania Dell'Orso, Zelalem Demere, Kush Mansuria, Carolina A. Mendonça, Octavio M. Palacios-Gimenez, Eli Ross, Max Savery, Kevin Yu, Harold E. Smith, Vittorio Sartorelli, Haiwang Yang, Nasser M. Rusan, Maria D. Vibranovski[†], Erika Matunis[†], and Brian Oliver[†^]

* These authors contributed equally

† These authors jointly supervised this work

^ Corresponding author

This PDF file includes:

Supplementary figures and legends, and dataset, file, and movie descriptions

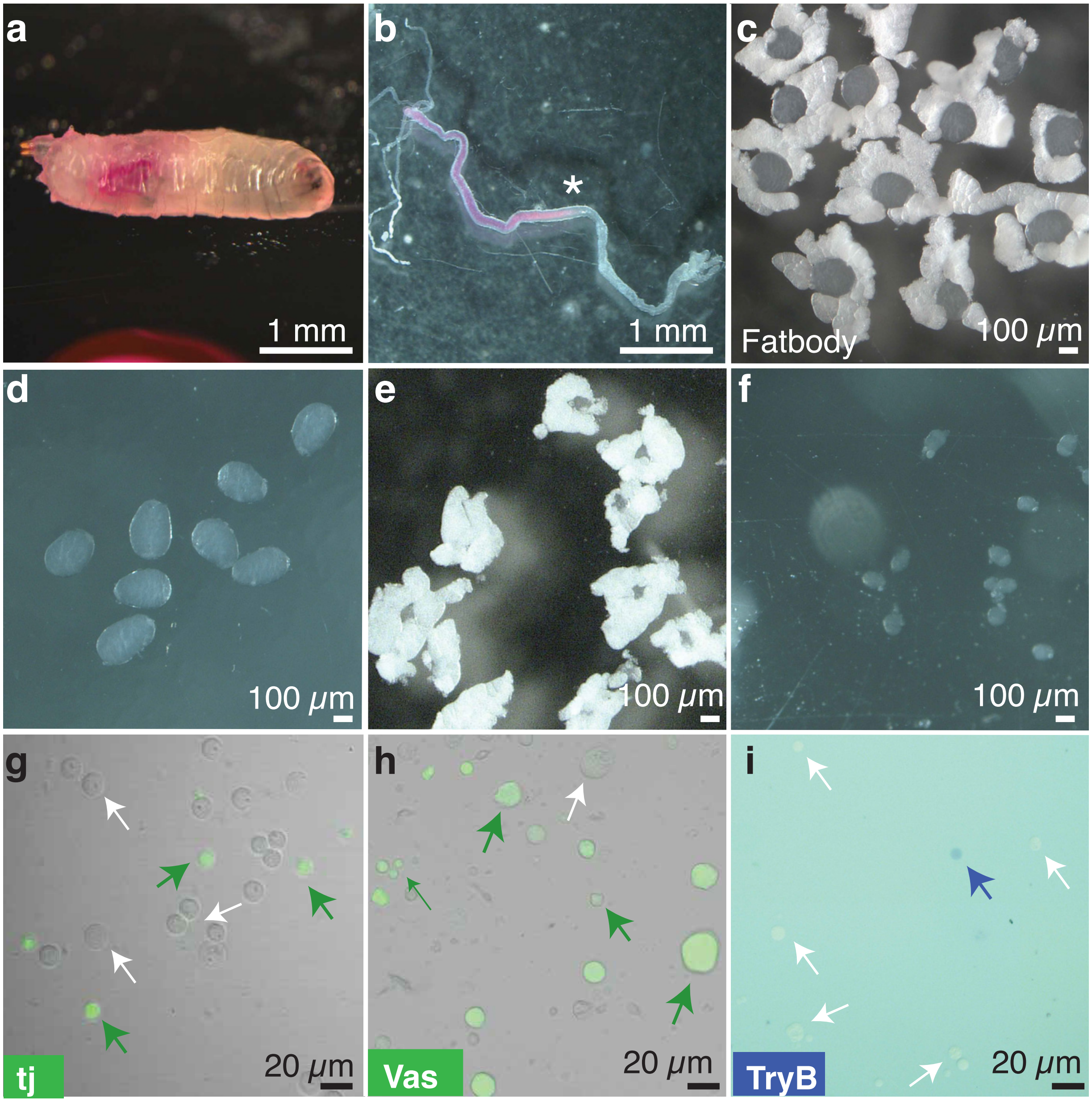

Supplemental Figure 1. Preparation of Drosophila Larval testis samples for scRNA-seq. (**a**) Bright-field microscopic image of late third instar larva fed with Sulforhodamine dye media (red). (**b**) Dissected larval gut with dye cleared from the foregut and remaining in the midgut and hindgut but clear foregut. The boundary of food clearing in the midgut (*). (**c**, **e**) Dissected larval testes and ovaries with fatbody (white). (**d**, **f**) Testes and ovaries cleaned of associated fatbody. Representative images (fluorescence and phase contrast) of dissociated cells marked for (**g**) *traffic jam* expression in the cyst cells (*tj-GAL4>UAS-GFP*) and (**h**) *vasa* expression in the germ cells (VASA-GFP), respectively (green arrows). Smaller *vasa* expressing cells (small green arrows). Cells not expressing GFP were also visible (white arrows). (**i**) Trypan blue (TryB) staining indicate dead (blue arrows) and live (white arrows) cells. White scale bars: **a-b** 1 mm, **c-f** 100 µm , Black scale bars: 20 µm.

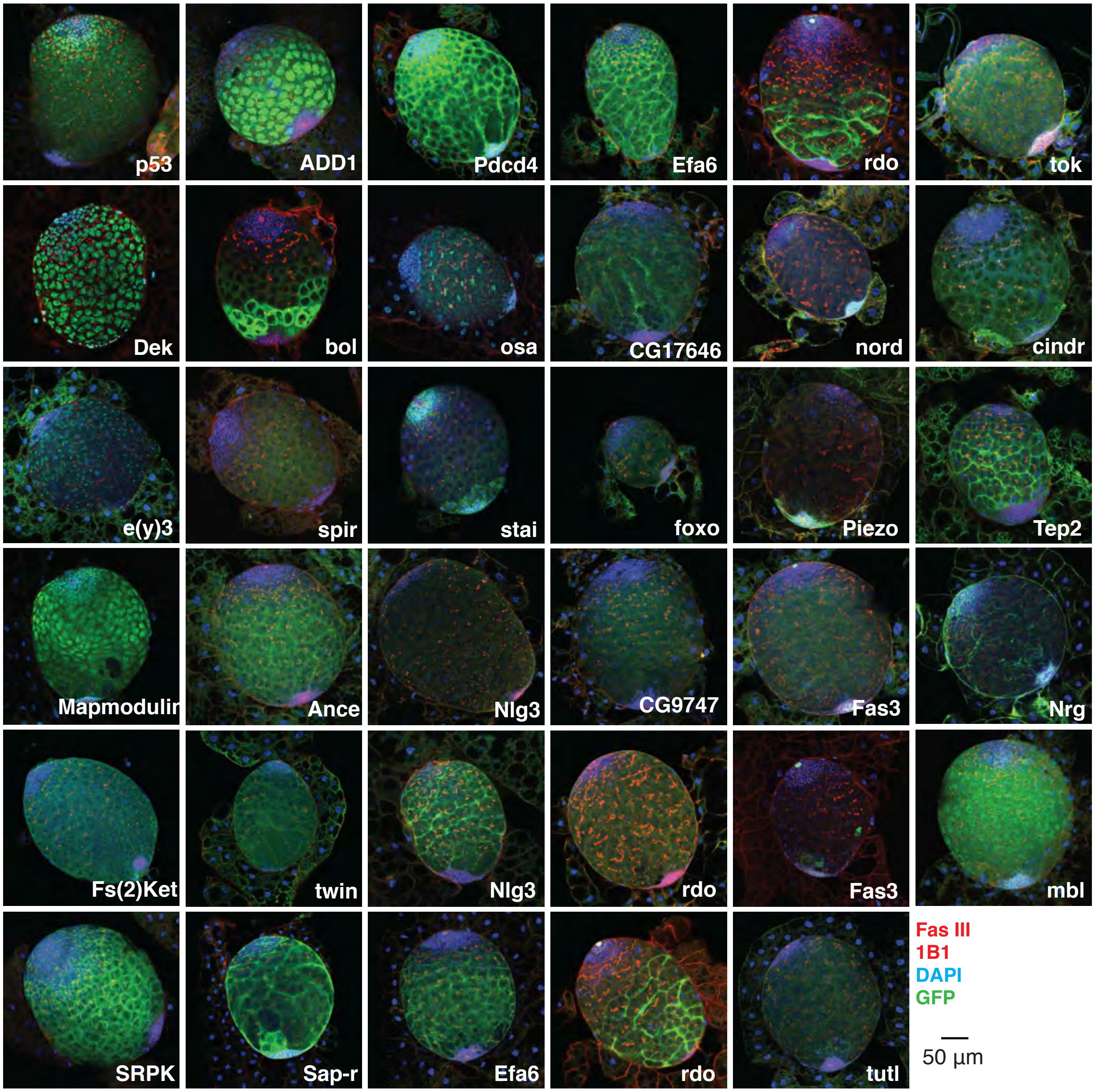

Supplemental Figure 2. Immunofluorescence of protein traps used to annotate cell clusters from scRNA-Seq data.

Immunofluorescence images showing protein expression of genes (green:GFP) from the protein trap reporters. Each panel consists of an individual gene (symbol) with color stained with anti-Fas III and 1B1 antibody (red), GFP (green) and counterstained with DAPI (blue). Black scale bar 50 µm.

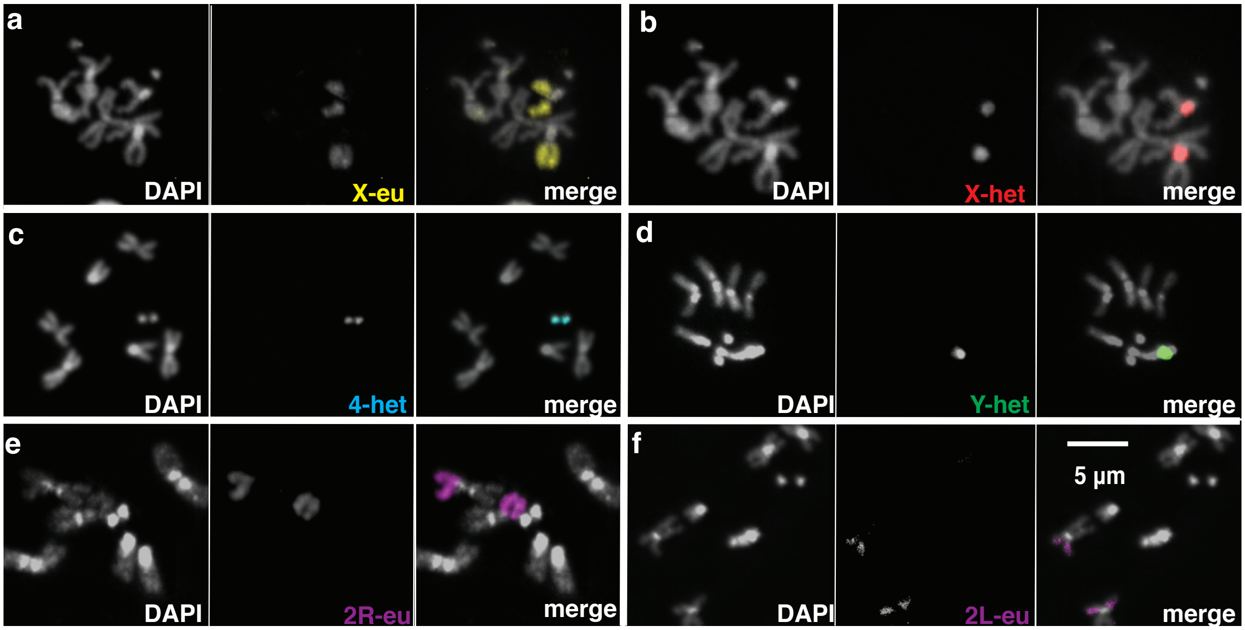

Supplemental Figure 3. Spatial location of chromosomal territories in the Metaphase chromosome spreads from the brain. (**a**) Euchromatin oligopaints (yellow) detected a pair of telocentric chromosomes in the genome indicating X euchromatin territory (**b**) 1.688 satellite probe (red) detect the same telocentric chromosome in the same nucleus as in A, indicating the X heterochromatin (**c**)AATAT probe detected a pair of dot chromosomes indicating the 4th heterochromatin (**d**) AATAC probe detect the single chromosome indicating the Y heterochromatin. Euchromatin oligopaints (purple) detect the large metacentric chromosomes in (**e**) and (**f**) indicating either 2R or 2L euchromatin that is indistinguishable clearly. Nuclei counterstained with DAPI (gray). White scale bar: 5 µm.

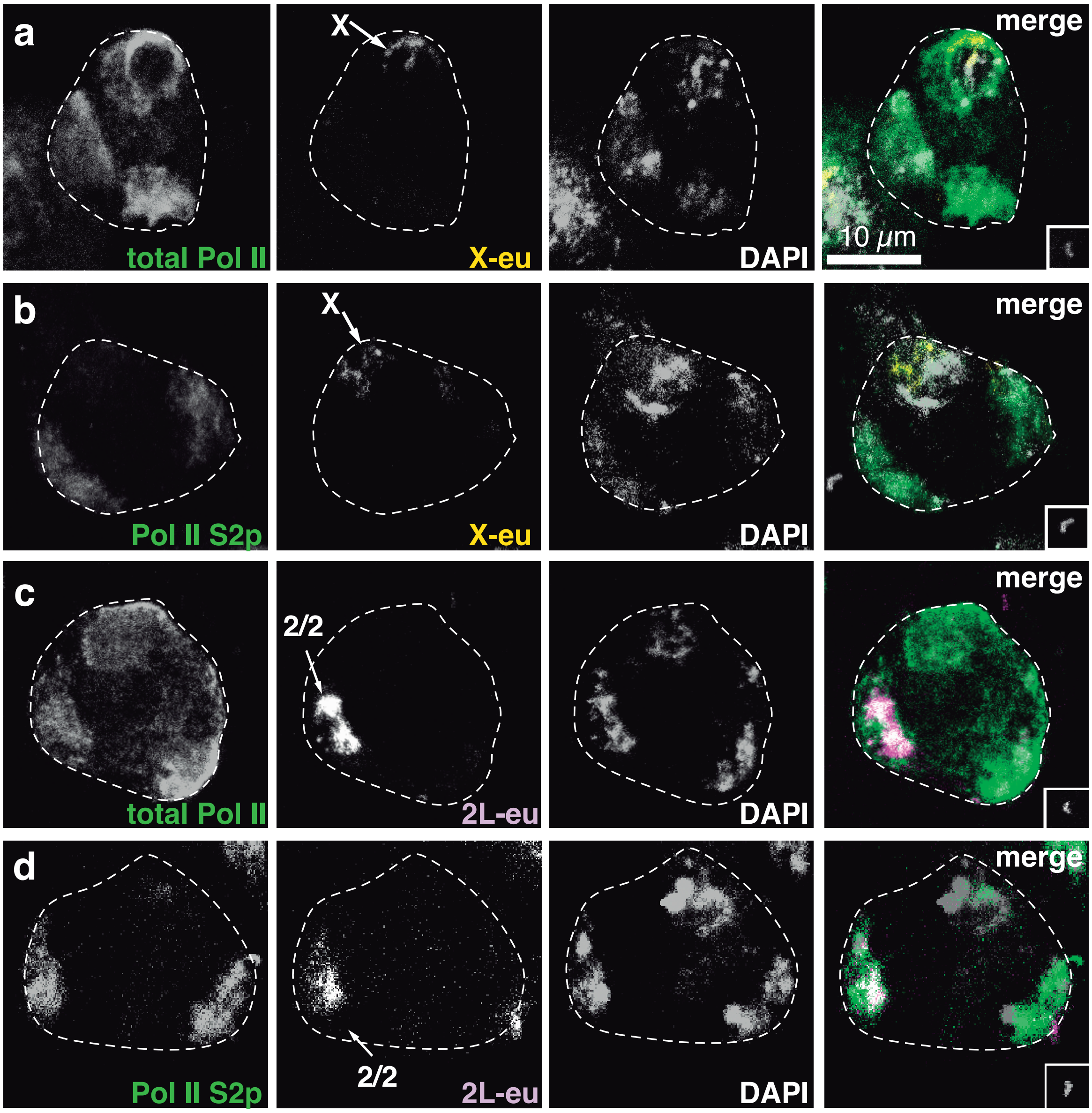

Supplemental Figure 4. Localization of Total Pol-II and pSer2-CTD on X and 2L co-stained with euchromatic oligopaints.

Spatial localization of X chromosome euchromatin by oligopaints (yellow) with (**a**) total RNA Polymerase-II (total Pol-II) and (**b**) Serine 2 phosphorylated Pol-II (Pol-II S2p). Spatial localization of 2L chromosome euchromatin by oligopaints (purple) with (**c**) total Pol-II and (**d**) (Pol-II S2p). DAPI (gray), primary spermatocyte nucleus is outlined (white-dashed line) based on DAPI and bright-field images (not shown). Asterless stained centrioles used to stage individual cells (insets). White scale bar: 10 μm.

Supplementary Datasets:

Supplemental Dataset 1. Bulk RNA-seq data. Consists of four parts: a readme, summary of gene expression and sex-biased significance in each sample set, Chi-square test of independence, and *post-hoc* tests to determine the significance of sex-biased gene expression by chromosome elements. Supporting data for Fig. 1 and Fig. 2c,d.

Supplemental Dataset 2. scRNA-seq data. Consists of 14 parts: a readme, cell level data, gene level data, chromosome level data, Y-chromosome expressed genes, cluster level data, one vs rest differential expression, spermatogonia versus spermatocyte analysis, spermatogonia versus early primary spermatocyte, early primary spermatocyte versus middle and late primary spermatocyte, early primary spermatocyte versus middle primary spermatocyte, early primary spermatocyte versus late primary spermatocyte, middle primary spermatocyte versus late primary spermatocyte, and Gene Set Enrichment Analysis for bulk RNA-Seq experiments. Supporting data for Fig. 2b-e.

Supplemental Dataset 3. Comparing bulk and single cell RNA-seq data. Consists of four parts: a readme, gene level raw data for bulk RNA-seq of adult and larval gonads, rank correlations among bulk RNA-seq and scRNA-seq samples, and rank correlations by testis cell type. Supporting data for Fig. 1k and Fig. 2c.

Supplemental Dataset 4. scRNA-seq cell type annotation. Consists of three parts: a readme, genes used for cell type annotation of single cell data, and cross tabulation of overlap with protein traps. Genes include re-curated images from the and reporter genes from the Cooley lab that are shown to be expression in specific cell types of the testis in this work (bold).

Supplemental Dataset 5. Gene sets and chromosome elements. Consists of six parts: a readme, distribution of genes among Chromosome elements, pairwise comparisons of global expression between different cell types using different gene sets (all expressed, CTSP, Tαυ, and TSPS), GO analysis of gene sets, X/A, 4/A, and Y/A ratio significance testing, pairwise significance testing for all chromosome elements by gene set. Supporting data for Table 1, Fig. 1b, Fig. 2e, and Fig. 4.

Supplemental Dataset. 6. FlyBase Author's Resources. This provides a compendium of details about stocks, reagents, software, vendors, sources, code, and datasets used in this project, and where to find them. Also used by FlyBase for data curation.

Supplementary Movie:

Supplemental Movie 1. X and 2L chromosome spatial localization in primary spermatocytes. Spatial location of X chromosome euchromatin by oligopaints (yellow) and 2L chromosome euchromatin by oligopaints (purple) within a primary spermatocyte. DAPI (gray), Asterless stained centrioles to stage individual cells (gray). Images are 'masked' in Imaris to obtain a 3D movie.
