# Supplementary material for: Dynamic sex chromosome expression in Drosophila male germ cells
Source: Nat Commun. 2021 Feb 9;12:892. doi: 10.1038/s41467-021-20897-y (PMC7873209; doi:10.1038/s41467-021-20897-y)
Supplement: Supplementary file 3 — Description of Additional Supplementary Files [file 41467_2021_20897_MOESM3_ESM.pdf]

Description of Additional Supplementary Files

Supplementary Dataset 1. Bulk RNA-seq data.

Consists of four parts: a readme, summary of gene expression and sex-biased significance in each sample set, Chi-square test of independence, and *post-hoc* tests to determine the significance of sex-biased gene expression by chromosome elements. Supporting data for **Fig. 1** and **Fig. 2c,d**.

Supplementary Dataset 2. scRNA-seq data.

Consists of 14 parts: a readme, cell level data, gene level data, chromosome level data, Y-chromosome expressed genes, cluster level data, one vs rest differential expression, spermatogonia versus spermatocyte analysis, spermatogonia versus early primary spermatocyte, early primary spermatocyte versus middle and late primary spermatocyte, early primary spermatocyte versus middle primary spermatocyte, early primary spermatocyte versus late primary spermatocyte, middle primary spermatocyte versus late primary spermatocyte, and Gene Set Enrichment Analysis for bulk RNA-Seq experiments. Supporting data for Fig. **b-e**.

Supplementary Dataset 3. Comparing bulk and single cell RNA-seq data.

Consists of four parts: a readme, gene level raw data for bulk RNA-seq of adult and larval gonads, rank correlations among bulk RNA-seq and scRNA-seq samples, and rank correlations by testis cell type. Supporting data for **Fig. 1k** and **Fig. 2c**.

Supplementary Dataset 4. scRNA-seq cell type annotation.

Consists of three parts: a readme, genes used for cell type annotation of single cell data, and cross tabulation of overlap with protein traps. Genes include re-curated images from the literature and reporter genes from the Cooley lab that are shown to be expression in specific cell types of the testis in this work (bold).

Supplementary Dataset 5. Gene sets and chromosome elements.

Consists of six parts: a readme, distribution of genes among Chromosome elements, pairwise comparisons of global expression between different cell types using different gene sets (all expressed, CTSP, Tau, and TSPS), GO analysis of gene sets, X/A, 4/A, and Y/A ratio significance testing, pairwise significance testing for all chromosome elements by gene set. Supporting data for **Table 1**, **Fig. 1b**, **Fig. 2e**, and **Fig. 4**.

Supplementary Dataset. 6. FlyBase Author's Resource Table.

This table provides a compendium of details about stocks, reagents, software, vendors, sources, code, and datasets used in this project, and where to find them. Also used by FlyBase for data curation.

Supplementary Movie 1. X and 2L chromosome spatial localization in primary spermatocytes.

Spatial location of X chromosome euchromatin by oligopaints (yellow) and 2L chromosome euchromatin by oligopaints (purple) within a primary spermatocyte. DAPI (gray), Asterless stained centrioles to stage individual cells (gray). Images are ‘masked’ in Imaris to obtain a 3D movie.
