# Supplementary material for: Dynamic sex chromosome expression in Drosophila male germ cells
Source: Nat Commun. 2021 Feb 9;12:892. doi: 10.1038/s41467-021-20897-y (PMC7873209; doi:10.1038/s41467-021-20897-y)
Supplement: Supplementary file 11 — Reporting Summary [file 41467_2021_20897_MOESM11_ESM.pdf]

nature research

Corresponding author(s): Brian Oliver

Last updated by author(s): YYYY-MM-DD

Reporting Summary

Nature Research wishes to improve the reproducibility of the work that we publish. This form provides structure for consistency and transparency in reporting. For further information on Nature Research policies, see our Editorial Policies and the Editorial Policy Checklist.

Statistics

For all statistical analyses, confirm that the following items are present in the figure legend, table legend, main text, or Methods section.

n/a	Confirmed	
☐	☒	The exact sample size (n) for each experimental group/condition, given as a discrete number and unit of measurement
☐	☒	A statement on whether measurements were taken from distinct samples or whether the same sample was measured repeatedly
☐	☒	The statistical test(s) used AND whether they are one- or two-sided<br>*Only common tests should be described solely by name; describe more complex techniques in the Methods section.*
☒	☐	A description of all covariates tested
☐	☒	A description of any assumptions or corrections, such as tests of normality and adjustment for multiple comparisons
☐	☒	A full description of the statistical parameters including central tendency (e.g. means) or other basic estimates (e.g. regression coefficient) AND variation (e.g. standard deviation) or associated estimates of uncertainty (e.g. confidence intervals)
☐	☒	For null hypothesis testing, the test statistic (e.g. F, t, r) with confidence intervals, effect sizes, degrees of freedom and P value noted<br>*Give P values as exact values whenever suitable.*
☒	☐	For Bayesian analysis, information on the choice of priors and Markov chain Monte Carlo settings
☒	☐	For hierarchical and complex designs, identification of the appropriate level for tests and full reporting of outcomes
☒	☐	Estimates of effect sizes (e.g. Cohen's d, Pearson's r), indicating how they were calculated

Our web collection on statistics for biologists contains articles on many of the points above.

Software and code

Policy information about availability of computer code

Data collection	All code is available is available at the Zendo repository: Doi:10.5281/zenodo.3973174
Data analysis	Commercial Software:<br>Imaris (v9.2.1)<br>Open Source Software:<br>Single cell selection: Cell Ranger v3.0.2, DropletUtils v1.2.1, Scrublet v0.2<br>Single cell analysis: Cell Ranger v3.0.2, Seurat v3.0.1<br>Bulk RNA-Seq analysis: bcl2fast v2.17.1.14, Hisat2 v.2.1.0, samtools v1.7, featureCounts v1.6.0, FASTQC v0.11.7, FastQ Screen v0.11.3, Picard v2.18.5, clusterProfiler v3.10.1<br>Statistical analysis and visualizations: Pandas v0.25.1, Scipy v1.3.1, Statsmodels v0.10.1, Matplotlib v3.1.0, Seaborn v0.9.0<br>Other: R v3.5.1, Python v3.7, Bioconda v5.5.0, Snakemake v3.5.1, Jupyter Notebook v6.0.1, Rstudio v1.1.423, Fiji v2.0.0<br>Custom software: All custom code is currently at https://github.com/jfear/larval_gonad project code will be uploaded to zenodo after paper revision.

For manuscripts utilizing custom algorithms or software that are central to the research but not yet described in published literature, software must be made available to editors and reviewers. We strongly encourage code deposition in a community repository (e.g. GitHub). See the Nature Research guidelines for submitting code & software for further information.

Data

Policy information about availability of data

All manuscripts must include a data availability statement. This statement should provide the following information, where applicable:

- Accession codes, unique identifiers, or web links for publicly available datasets
- A list of figures that have associated raw data
- A description of any restrictions on data availability

All data is available in the main text, the supplementary materials, and/or at NCBI Gene Expression Omnibus (GSE125947, GSE115511, and GSE115478).
Figures 2, 4, 5, 6 S1, S3, S4, and S6 contain raw data.
There are no restrictions on data availability.

Field-specific reporting

Please select the one below that is the best fit for your research. If you are not sure, read the appropriate sections before making your selection.

☒ Life sciences ☐ Behavioural & social sciences ☐ Ecological, evolutionary & environmental sciences

For a reference copy of the document with all sections, see nature.com/documents/nr-reporting-summary-flat.pdf

Life sciences study design

All studies must disclose on these points even when the disclosure is negative.

Sample size	No sample-size calculation was performed statistically.<br>All sample sizes are comparable or exceed current sample sizes used in the literature.<br>For single cell RNA-Seq,three biological replicates were chosen as there was high sample correlation when one or three samples were chosen.<br>Most of the single cell studies in literature used no replicates.
Data exclusions	We excluded protein traps that had little or no expression. This exclusion criteria was predetermined.
Replication	Within an experiment, samples were compared using Spearman correlations. We also compared bulk RNA-Seq with scRNA-Seq to verify reproducibility across technologies.
Randomization	Randomization is not relevant to this study because there are no case/control type comparisons. All comparisons are within individual cells or within a single biological class
Blinding	Blinding was not relevant to this study because there are no case/control type comparisons.

Reporting for specific materials, systems and methods

We require information from authors about some types of materials, experimental systems and methods used in many studies. Here, indicate whether each material, system or method listed is relevant to your study. If you are not sure if a list item applies to your research, read the appropriate section before selecting a response.

Materials & experimental systems

n/a	Involved in the study	
☐	☒	Antibodies
☒	☐	Eukaryotic cell lines
☒	☐	Palaeontology and archaeology
☐	☒	Animals and other organisms
☒	☐	Human research participants
☒	☐	Clinical data
☒	☐	Dual use research of concern

Methods

n/a	Involved in the study	
☒	☐	ChIP-seq
☒	☐	Flow cytometry
☒	☐	MRI-based neuroimaging

Antibodies

Antibodies used	mouse Anti-RNA polymerase II, clone CTD4H8 MilliporeSigma, Burlington, MA, gift from Eduardo Gorab Millipore05-623; RRID:AB_309852<br>rat Anti-RNA polymerase II subunit B1 (phospho CTD Ser-2), clone 3E10 antibody MilliporeSigma, Burlington, MA Millipore:04-1571; RRID:AB_11212363

rabbit Anti-RNA polymerase II CTD repeat YSPTSPS (phospho S5) Abcam, Cambridge, MA Abcam:ab5131; RRID:AB449369
guinea pig Anti-Asl A gift of G. Rogers, University of Arizona, Tucson, AZ
Mouse Anti-Lamin DmO DSHB, Iowa City, IA DSHB:ADL84.12; RRID:AB_528338
Mouse Anti-Lamin C DSHB, Iowa City, IA DSHB:LC28.26; RRID:AB_528339
Mouse Anti-Fasciclin III DSHB, Iowa City, IA DSHB:7G10; RRID:AB_528070
Mouse Anti-hu-li tai shao, not hts-PC DSHB, Iowa City, IA DSHB:1B1; RRID:AB_528070
Goat anti-Guinea Pig IgG (H+L) - Alexa Fluor 488 Thermo Fisher Scientific, Waltham, MA Invitrogen:A11073; RRID:AB_2534117
Goat anti-Mouse IgG (H+L) - Alexa Fluor 568 Thermo Fisher Scientific, Waltham, MA Invitrogen:A11004; RRID:AB_2534072
Goat anti-Rabbit IgG (H+L) - Alexa Fluor 647 Thermo Fisher Scientific, Waltham, MA Invitrogen:A21244; RRID:AB2535812
Goat anti-Rat IgG (H+L) - Alexa Fluor 647 Thermo Fisher Scientific, Waltham, MA Invitrogen:A21247; RRID:AB_141778
Goat anti-Chicken IgY (H+L) - Alexa Fluor 488 Thermo Fisher Scientific, Waltham, MA Invitrogen:A11039; RRID:AB_2534096
Goat anti-Mouse IgG (H+L) - Alexa Fluor 488 Thermo Fisher Scientific, Waltham, MA Invitrogen:A11029; RRID:AB_2534088
Chicken Anti-GFP Abcam, Cambridge, MA Abcam:ab13970; RRID:AB_300798
Rabbit Anti-dimethyl-Histone H3 (Lys9) MilliporeSigma, Burlington, MA Millipore:07-441; RRID:AB_310619
Rabbit Anti-acetyl-Histone H4 (Lys5) MilliporeSigma, Burlington, MA Millipore:07-327; RRID:AB_310523
Goat Anti-Rabbit IgG (whole molecule)–FITC MilliporeSigma, Burlington, MA Sigma:F0382; RRID:AB_259384
Sheep Anti-digoxigenin-rhodamine, Fab fragments Roche, Mannheim, Germany Roche:11 207 750 910

Validation

Antibodies are validated by the supplier by either immunoprecipitation by a cell lysate and analyzed by mass spectrometry, western blot and detecting the protein in tissue or cells.

Animals and other organisms

Policy information about studies involving animals; ARRIVE guidelines recommended for reporting animal research

Laboratory animals

All strains used L3 larval or adult males. We also use data from adult w[1118] females.
y w
Tempe-T
w[1118]
w[1118] P{w[+mC]=PTT-GA}Nrg[G00305]
w[1118]; Mi{ET1}fln[MB03038]/TM3, Sb[1] Ser[1]
“y[1] w[*]; Mi{y[+mDint2]=MIC}rdo[MI02219]”
“w[*]; P{w[+mC]=PTT-un}Fs(2)Ket[GFP]/CyO; P{w[+mC]=mRFP-Nup107.K}7.1”
“y[1] w[*]; Mi{y[+mDint2]=MIC}Mlc2[MI02834]”
“y[1] w[*]; Mi{y[+mDint2]=MIC}Nlg3[MI02443]/TM3, Sb[1] Ser[1]”
“y[1] w[*]; Mi{y[+mDint2]=MIC}Cht5[MI03431]/TM3, Sb[1] Ser[1]”
w[1118] P{w[+mC]=PTT-GB}Pdcd4[G93]
“y[1] w[*]; Mi{PT-GFSTF.1}tutl[MI00290-GFSTF.1]/SM6a”
“y[1] w[*]; Mi{y[+mDint2]=MIC}Syn[MI05723]”
y[1] w[*] Mi{y[+mDint2]=MIC}RunxA[MI07404]
“y[1] w[*]; P{w[+mC]=PTT-GA}cindr[CA06686]”
w[*]; P{w[+mC]=PTT-un}Dek[G00131]
“w[*]; P{w[+mC]=PTT-GA}Fas3[G00258]”
“y[1] w[*]; Mi{y[+mDint2]=MIC}rdo[MI08797]”
w[*] P{w[+mC]=PTT-GC}e(y)3[CC01368]
“w[*]; P{w[+mC]=PTT-GC}Mapmodulin[CC01398]”
“w[*]; P{w[+mC]=PTT-GC}osa[CC00445]”
y[1] w[*]; Mi{y[+mDint2]=MIC}rdo[MI10011]”
“y[1] w[*]; Mi{y[+mDint2]=MIC}Syn[MI11740]”
“y[1] w[*]; Mi{y[+mDint2]=MIC}Nlg3[MI08924]”
“y[1] w[*]; Mi{y[+mDint2]=MIC}sosie[MI12650]”
“y[1] w[67c23]; Mi{PT-GFSTF.2}Tep2[MI01299-GFSTF.2]”
“y[1] w[*]; Mi{y[+mDint2]=MIC}cmpy[MI14851]”
“y[1] w[67c23]; Mi{PT-GFSTF.0}mbl[MI00139-GFSTF.0]/CyO”
“y[1] w[*]; Mi{PT-GFSTF.0}foxo[MI00493-GFSTF.0]/TM6C, Sb[1] Tb[1]”
“y[1] w[67c23]; Mi{PT-GFSTF.1}Fas3[MI03674-GFSTF.1]/SM6a”
“y[1] w[67c23]; Mi{PT-GFSTF.0}spir[MI05646-GFSTF.0]/CyO”
“y[1] w[67c23]; Mi{PT-GFSTF.1}Ance[MI05748-GFSTF.1]/SM6a”
“y[1] w[*]; Mi{PT-GFSTF.0}Efa6[MI00261-GFSTF.0]/TM6C, Sb[1] Tb[1]”
“y[1] w[*]; Mi{PT-GFSTF.1}CG9747[MI02072-GFSTF.1]/TM3, Sb[1] Ser[1]”
“y[1] w[67c23]; Mi{PT-GFSTF.0}Piezo[MI04189-GFSTF.0]”
“y[1] w[67c23]; Mi{PT-GFSTF.2}nord[MI06414-GFSTF.2]/SM6a”
“y[1] w[*]; Mi{PT-GFSTF.0}p53[MI01307-GFSTF.0]/TM6C, Sb[1] Tb[+]”
“y[1] w[*]; Mi{PT-GFSTF.1}tok[MI06118-GFSTF.1]/TM6C, Sb[1] Tb[1]”
“y[1] w[67c23]; Mi{PT-GFSTF.1}ADD1[MI09552-GFSTF.1]/CyO”
“w[*]; TI{TI}Efa6[GFP-C]”
“y[1] w[*]; Mi{y[+mDint2]=MIC}kkv[MI15197] CG14668[MI15197]/TM3, Sb[1] Ser[1]”
“y[1] w[*]; Mi{PT-GFSTF.1}dpr17[MI08707-GFSTF.1]”
“y[1] w[*]; Mi{PT-GFSTF.1}twin[MI07336-GFSTF.1]/TM6C, Sb[1] Tb[1]”
“y[1] w[*]; Mi{PT-GFSTF.2}Sap-r[MI11015-GFSTF.2]/TM6C, Sb[1] Tb[1]”
“y[1] w[*]; Mi{PT-GFSTF.1}bol[MI00386-GFSTF.1]/TM6C, Sb[1] Tb[1]”
“y[1] w[*]; Mi{PT-GFSTF.0}CG17646[MI04004-GFSTF.0]”
“y[1] w[*]; Mi{PT-GFSTF.1}SRPK[MI06550-GFSTF.1]”

“y[1] w[*]; Mi{PT-GFSTF.1}stai[MI07398-GFSTF.1]”
“P{GawB}E132; ; P{w[+mC]=tubP-GAL80[ts]}2”
“y[*] w[*]; P{w[+mC]=UAS-2xEGFP}AH3”
“y[*] w[*]; P{GawB}NP1624 / CyO, P{UAS-lacZ.UW14}UW14”
“w[*]; P{vas.EGFP.HA}”

Wild animals	This study did not involve wild animals.
Field-collected samples	Tempe-T flies are collected from the field
Ethics oversight	This study only used Drosophila melanogaster, which does not require ethics oversight.

Note that full information on the approval of the study protocol must also be provided in the manuscript.
